# Activation of Wnt/ß-catenin signaling in ESC promotes rostral forebrain differentiation in vitro

**DOI:** 10.1007/s11626-015-9975-y

**Published:** 2015-11-12

**Authors:** Nozomu Takata, Eriko Sakakura, Yoshiki Sasai

**Affiliations:** Laboratory for in vitro Histogenesis, RIKEN Center for Developmental Biology, 2-2-3 Minatojima-minamimachi, Chuou-ku, Kobe, Hyogo 650-0047 Japan; Laboratory for Organogenesis and Neurogenesis, RIKEN Center for Developmental Biology, 2-2-3 Minatojima-minamimachi, Chuou-ku, Kobe, Hyogo 650-0047 Japan

**Keywords:** Wnt/ß-catenin signaling, Wnt antagonist, Rostral forebrain, Rax, Embryonic stem cells, Regenerative medicine

## Abstract

Wnt/ß-catenin signaling is crucial for maintenance of pluripotent state of embryonic stem cell (ESC). However, it is unclear how Wnt/ß-catenin signaling affects the differentiation ability of ESC, especially with regard to rostral forebrain cells. Here, using Rax, rostral forebrain marker, and Wnt/ß-catenin reporter lines, we report ratio of Rax^+^ and Wnt responding tissue (Wnt^+^) patterns, which were affected by seeding number of ESC in three-dimensional culture system. Surprisingly, we found ß-catenin level and localization are heterogeneous in ESC colony by immunostaining and time-laps imaging of ß-catenin-mEGFP signals. Moreover, activation of Wnt signaling in ESC promoted expression level and nuclear localization of ß-catenin, and mRNA levels of Wnt antagonists, *axin2* and *dkk1*, leading to upregulating Wnt/ß-catenin reporter in ESC state and Rax expression at differentiation culture day 7. Together, our results suggest that activation of Wnt signaling in ESC promotes the differentiation efficacy of rostral forebrain cells. Wnt-priming culture method may provide a useful tool for applications in the areas of basic science and molecular therapeutics for regenerative medicine.

**Electronic supplementary material:**

The online version of this article (doi:10.1007/s11626-015-9975-y) contains supplementary material, which is available to authorized users.

## Introduction

Forebrain is derived from neural plate, called neural ectoderm (Ruiz i Altaba 1998), which is subdivided into telencephalon and diencephalon (Markakis 2002). Diencephalon contains the hypothalamus and eyes in vertebrate (Sinn and Wittbrodt 2013). These developmental events involving forebrain patterning are largely conserved among species (Wilson and Houart 2004).

In rostral forebrain cells, Rax, transcriptional factor is expressed during early development (Furukawa *et al.*^1997^; Ohuchi *et al.*^1999^). In fact, disruption of Rax gene led to severe defects involving forebrain and eye development (Mathers and Jamrich 2000; Zhang *et al.*^2000^; Andreazzoli *et al.*^2003^; Nakagawa 2004). Additionally, misexpression of Rax gene led to duplication of rostral neural tube and eye (Mathers *et al.*^1997^). Moreover, genetic mutations of the human RAX were found in a patient with anophthalmia and microphthalmia, which were ocular birth defects and a cause of congenital blindness (Voronina *et al.*^2004^; Abouzeid *et al.*^2012^). Thus, Rax gene plays crucial roles in forebrain and eye development.

Although its roles are well know as described above, Rax gene has another role as a specific marker for forebrain development. In this regard, for regenerative medicine, monitoring of Rax gene expression is a powerful tool for estimation of forebrain differentiation in vitro.

Previously, using embryonic stem cell (ESC) culture system, Wataya et al. showed efficient induction of hypothalamic tissues expressing Rax gene (Wataya *et al.*^2008^), and Eiraku et al. showed optic cup morphogenesis by monitoring Rax expression in live condition (Eiraku *et al.*^2011^). These reports demonstrated clearly that the use of Rax gene monitoring facilitated the analysis of forebrain development, suggesting that innovation of generation and manipulation of such tissues make it possible for regenerative tools to become closer to reality. However, in both methods, Rax expression patterns slightly vary among tissues in vitro (high and low expressions among tissues). Lacking of efficient and stable culture methods may hamper the reliability of future regenerative approaches.

In another aspect of ESC culture system, ESC state is maintained by Wnt signaling activity (Nusse 2008; Nusse *et al.*^2008^; ten Berge *et al.*^2011^). Namely, activation of the Wnt signaling maintains ESCs in a pluripotent state. In addition, ESCs that harbor Wnt activating mutations in the negative regulator APC or in positive regulator ß-catenin indeed have a profound reduction in their ability to differentiate, even following LIF (a ESC maintenance factor) withdrawal (Kielman *et al.*^2002^). Wnt signaling also regulates the lineage differentiation potential of ESCs (Atlasi *et al.*^2013^; Price *et al.*^2013^). 2i conditioned medium (ESC medium containing Wnt agonist, CHIR99021 (CHIR) and Fgf antagonist, PD0325901 (Ying *et al.*^2008^; Li and Ding 2010; Plusa and Hadjantonakis 2014)) improves ESC differentiation rate of neuronal lineages, expressing pan-neural marker, *sox1* (Marks *et al.*^2012^). Therefore, Wnt signaling in ESC maintenance is important for a potential lineage commitment. However, it is unclear how Wnt activation in ESC state affects rostral forebrain differentiation in vitro.

Here, we observed Rax gene expression with Wnt/ß-catenin signaling to explore the efficient method, which potentially promotes rostral forebrain differentiation from mouse ESCs.

## Materials and Methods

*ESCs maintenance*, *differentiation*, *and Wnt*-*priming method*. Mouse ESCs; E14Tg2a (Hooper *et al.*^1987^), Rax::GFP (Wataya *et al.*^2008^), and Rax::GFP//TOP::DsRed (Andrabi *et al.*^2015^) murine ESCs were maintained, and SFEBq culture were performed as described thoroughly by Wataya et al. (2008). Briefly, SFEBq (serum-free floating culture of embryoid body-like aggregates with quick reaggregation) culture is a method that starts with quick reaggregation of dissociated mouse ESCs in each well of a low cell-adhesive coating 96-well plate (Nunclon Sphera 96U Bottom Plate 174925; Thermo Scientific, Waltham, MA). For differentiation medium, we used gfCDM (a modified growth factor free chemically defined medium (Wiles and Johansson 1999; Bouhon *et al.*^2005^)), which is free of knockout serum replacement and other growth factors including insulin. Composition of gfCDM was as follows: Iscove’s modified Dulbecco’s medium (Gibco, Carlsbad, CA) and Ham’s F-12 Nutrient mixture (Gibco) are mixed in a one-to-one, supplemented with 1x chemically defined lipid concentrate (Gibco), monothioglycerol (450 μM), purified BSA (5 mg/ml), and human apo-transferrin (15 μg/ml). In Wnt-priming method, we maintained ESCs with 10 μM CHIR99021 (Stemgent, Cambridge, MA) for 2 or 3 d and then performed SFEBq differentiation in gfCDM. This method has one advantage, because it is not necessary to add additional chemical reagents during forebrain differentiation, meaning not necessary to remove chemical reagents completely (when collecting forebrain tissues for regenerative medicine). Differentiation status was quickly checked by BZ-9000 microscope (KEYENCE, Osaka, Japan), which allows us to monitor fluorescent and transillumination images.

*FACS analysis*. Fluorescence-activated cell sorting (FACS) analysis was performed as previously described (Kamiya *et al.*^2011^). For cell preparation, cells were dissociated to single cells by TrypLE™ Express (Gibco, 12605–010) treatment and filtration through Cell Strainer (BD Biosciences, East Rutherford, NJ). Cells in tube were kept on ice until analysis. For population analysis, FACSDiva (BD Biosciences) was used. For data analysis, data were analyzed with FlowJo software. All processes were performed, based on manufacturer’s instruction.

*Immunohistochemistry*, *confocal microscope*, *and image analysis*. Immunostaining and cryosectioning were performed as previously described (Wataya *et al.*^2008^; Ohgushi *et al.*^2015^). Briefly, for ESC staining, the ESCs were seeded onto a gelatin-coated 8-well chamber slide (Biocoat Collagen TypeI Cellware 8well Culture Slide 354630; CORNING, Corning, NY), and fixed with 4% paraformaldehyde (PFA) at room temperature for 30 min and then permeabilized with 0.3% Triton-X100/PBS solution. After incubation of ESCs in 2% skim milk/PBS blocking solution, we used specific antibodies in blocking solution as follows: ß-catenin (mouse, 1:500, BD transduction 610153: rabbit, 1:500, C-2206; Sigma-aldrich): GFP (rat, 1/500, 04404–84; Nacalai, San Diego, CA): Nanog (rabbit, 1/1000, RCAB0001P; ReproCell, Yokohama, Japan): Oct3/4 (mouse, 1/200, BD 611202). After washing primary antibodies in 0.05% Tween/PBS, the staining was visualized using appropriate secondary antibodies conjugated with the fluorescent probes, Alexa Fluor-488 (1:1000, Molecular Probes, Eugene, OR) or Cy3/Cy5 (1:200, Jackson ImmunoResearch, West Grove, PA). We used DAPI (#11034-56, Nacalai) for counter staining. F-actin was visualized with AlexaFluor-conjugated phalloidin (546 and 647, 1/500, A22283 and A22287, respectively; Invitrogen, Carlsbad, CA). For cryosectioned sample staining, day-7 tissues were fixed with 4% PFA at room temperature for 30 min and washed in PBS. Then fixed tissues were in 15% sucrose/PBS at 4°C for overnight for cryoprotection, followed by cryosection. Immunostaining of cryosectioned sample slides is basically the same as above. Imaging analysis was performed using LSM 710 or 780 confocal laser-scanning microscope (Zeiss, Oberkochen, Germany). The Z-scanning images in video 1 were reconstituted from the serial slices of confocal images using imageJ software.

*Rosa26 locus targeting vector construction*, *introduction*, *and genotyping of ß*-*catenin*-*mEGFP knock*-*in ESCs*. For Rosa26 locus knock-in, we obtained Rosa26 mT/mG (Muzumdar *et al.*^2007^), a gift from Liqun Luo (plasmid # 17787; Addgene, Cambridge, MA) and performed chimeric-PCR to amplify ß-catenin-mEGFP-SV40pA from mouse ESC cDNA and pDONR221-mEGFP (monomeric EGFP). Subsequently, by restriction enzymes, we subcloned ß-catenin-mEGFP-SV40pA PCR products into downstream of pCAG (consisting of the cytomegalovirus enhancer fused to the chicken beta-actin promoter) in Rosa26 mT/mG as a backbone vector, which has 5′ (1.1 kbp) and 3′ (4.3 kbp) homology arms with pPGK::neo^r^-bGHpA (mT/mG cDNA was removed at this time). Then, we linearized Rosa26 ß-catenin-mEGFP targeting vector by a restriction enzyme and purified it by phenol-chloroform extraction to eliminate enzyme proteins. Using electroporation grade of linearized vector (7.5 μg), we introduced it into 1.0×10^6^ E14Tg2a line via A24 program of Amaxa Nucleofector (Lonza, Basel, Switzerland) based on the manufacturer’s instruction (day 0). Next at day 2, we add 200 μg/ml G418 sulfate (10131–035, Gibco) to select pooled homologous recombinant ESCs. Finally, at day 10, we extracted genome of pooled-drug-resistant ESCs and performed genotyping of ß-catenin-mEGFP knock-in allele by primers (rosa3, 5′-CCACTGACCGCACGGGGATTC-3′; rosa4, 5′-TCAATGGGCGGGGGTCGTT-3′). We used pooled knock-in ESCs for Figs. 3 and 4.

*Time*-*laps imaging and analysis*. Live-imaging was performed using an incubator-combined confocal optic system (Olympus, Tokyo, Japan) as previously described (Eiraku *et al.*^2011^) using a thin plastic-bottom dish (μ-Dish, 35 mm, low; ibidi, 80136), supplying penicillin/streptomycin and then filmed using a LCV110 equipped with 488-nm excitation lasers. We edited acquired images using MetaMorph software (Molecular Devices, Sunnyvale, CA) and Image J (free software).

*RNA extraction*, *cDNA synthesis*, *and RT*-*qPCR*. RNA was extracted using the Quiacube (Qiagen, Hilden, Germany) using the company-provided protocol. As previous report (Suga *et al.*^2011^), 350 μl buffer RLT were added to dissociated ESC pellets and spun through QIAshredder (Qiagen) prior to RNA extraction. The cDNA samples for RT-qPCR reactions were generated using the SuperScriptII (18064–014, Invitrogen). The qPCR reactions were performed using a 7500 Fast Real-Time PCR System (Applied Biosystems, Foster City, CA): Standard curves were estimated using a series of dilutions of cDNA purified from mouse ESCs. Primer sets for qPCR are as follows:

**Table:** 

Gene	Forward	Reverse
GAPDH	TGACCACAGTCCATGCCATC	GACGGACACATTGGGGGTAG
*axin2*	TGACTCTCCTTCCAGATCCCA	TGCCCACACTAGGCTGACA
*dkk1*	CCGGGAACTACTGCAAAAAT	CCAAGGTTTTCAATGATGCTT

*Statistics*. Statistical analyses were performed with Prism (GraphPad Software, Inc., La Jolla, CA). Data sets were first checked for standard error of the mean. The appropriate test for comparison was performed as follows; *t* tests (two samples) and Dunnett’s test (control and others) were used to generate *P* values (*P < 0.05; **P < 0.01; ***P < 0.001).

## Results and Discussion

In this study, we developed a new method for efficient differentiation of Rax^+^ rostral forebrain by activating Wnt signaling through ß-catenin stabilization in mouse ESCs.

To date, the level of Wnt signaling activity in individual stem cells correlates with differences in lineage-specific differentiation propensity (Blauwkamp *et al.*^2012^). Here, we endeavored to extend the idea involving Wnt signaling/ß-catenin in ESC state and its roles for differentiation, especially in rostral neural lineage.

**Figure 1.**
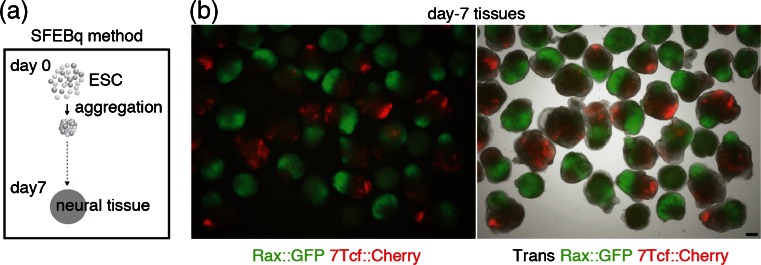
Rostral forebrain marker, Rax::GFP and Wnt reporter, 7Tcf::Cherry expressed in SFEBq-derived tissues. (*a*) Schematic diagram of SFEBq method. (*b*) Merged images of transillumination (Trans), Rax::GFP and 7Tcf::Cherry signals in day-7 tissues. *Scale bars* 100 μm.

**Figure 2.**
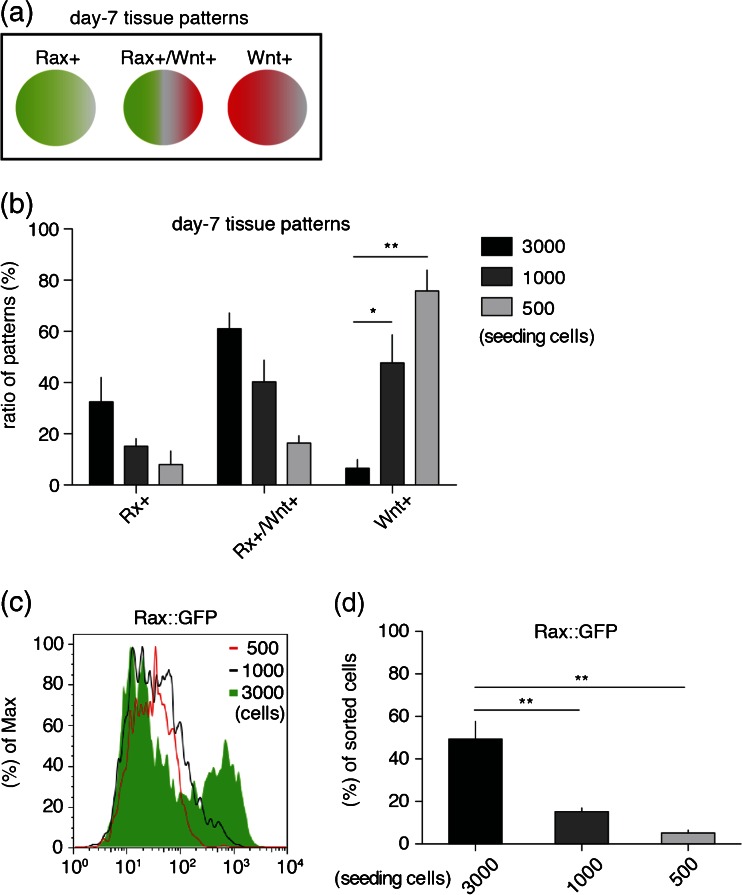
Seeding number in SFEBq affected the expression patterns of Rax::GFP and 7Tcf::Cherry signals. (*a*) Schematic diagram of day-7 tissue patterns. (*b*) Quantification of ratio of day-7 tissue patterns in several seeding number condition. *Error bars* indicate standard error of the mean of each experiment (*N* = 96 tissues). (*c*) FACS analysis of day-7 Rax::GFP positive cells in several seeding number condition. (*d*) Quantification of Rax::GFP expression of day-7 cells. *Error bars* indicate standard error of the mean of 16 tissues of each experiment.

*Rostral forebrain differentiation was accompanied by Wnt signaling and seeding cell number in SFEBq affected Rax*^+^/*Wnt*^+^*tissue patterning*. We first endeavored to analyze Wnt signaling in rostral forebrain differentiation, using SFEBq, which enable us to observe tissue differentiation like that in vivo in three-dimensional (3D) manner (Fig. 1*a*; see “Materials and Methods” section). We utilized Rax::GFP//7Tcf::Cherry line to monitor rostral forebrain and Wnt responding tissues during differentiation and found day-7 tissues showed several Rax::GFP^+^ and 7Tcf::Cherry^+^ expression patterns among individual tissues (Fig. 1*b*). The types of expression patterns of tissues were classified into three types as follows: Rax+; most cells expressed Rax::GFP^+^, Rax+/Wnt+; Both Rax::GFP^+^ and 7Tcf::Cherry^+^ patterns within each tissue and Wnt+; Majority of cells are 7Tcf::Cherry^+^ or Rax::GFP^−^ cells (Fig. 2*a*). These results imply Wnt signaling is involved in tissue pattern formation and are consistent with previous reports (ten Berge *et al.*^2008^; Petersen and Reddien 2009).

Next, we tested weather seeding number of cells in SFEBq affect the expression patterns of Rax::GFP^+^ and 7Tcf::Cherry^+^. Compared with 3000 cells experimental condition, we found that decreasing seeding cell number significantly increased Wnt^+^ ratio but decrease Rax^+^ ratio as seen in 1000 and 500 cells (Fig. 2*b*). Furthermore, via FACS analysis, Rax::GFP^+^ cells were significantly decreased in 1000 and 500 cells condition (Fig. 2*c*, *d*), indicating that population of Rax^+^ rostral forebrain cells are correlated inversely with Wnt signals and proportionally with seeding cell numbers.

**Figure 3.**
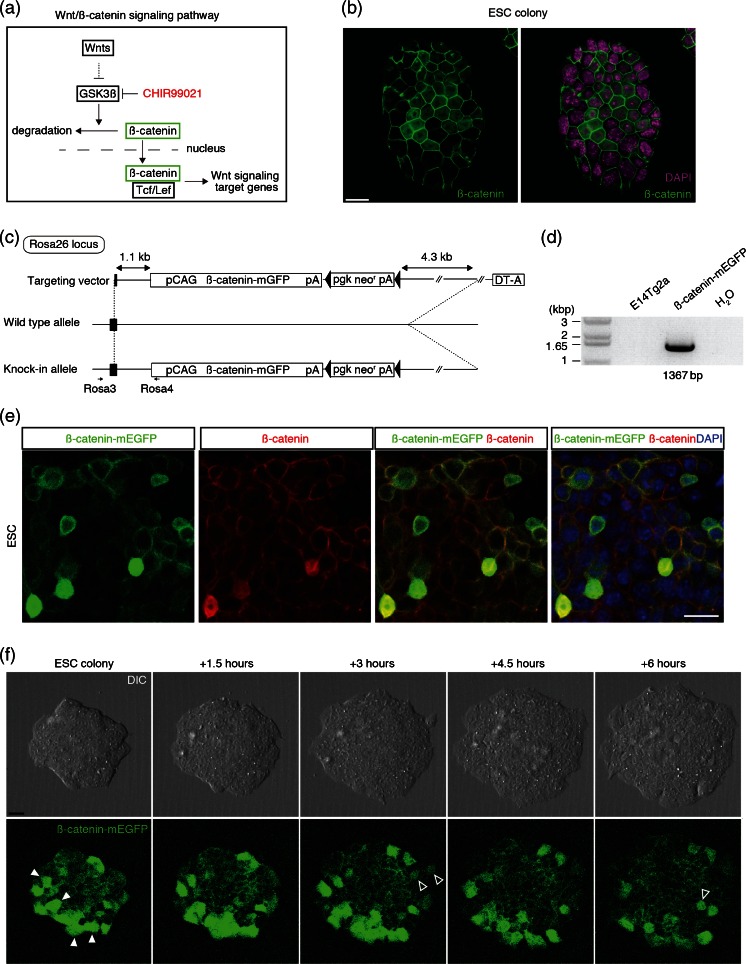
Wnt downstream mediator; ß-catenin was heterogeneously expressed in individual ESCs. (*a*) Diagram of Wnt/ß-catenin signaling. (*b*) Immunohistochemistry was performed on ESC colonies with DAPI using antibodies recognizing ß-catenin. (*c*) Diagram of Rosa 26 locus knock-in by ß-catenin-mEGFP targeting vector. *Black box*, exon 1, *pA* poly adenylation signal, *pgk* promoter PGK, *neo* ^*r*^ neomycin resistant gene, *DT*-*A* A subunit of diphtheria toxin gene under control of PGK promoter, *black triangles* Cre-mediated site specific sequence for recombination. (*d*) PCR detection of ß-catenin-mEGFP knocked-in allele. *Left lane* is DNA ladder marker. E14Tg2a is a parental line. (*e*) Immunostaining of ß-catenin-mEGFP knocked-in ESC using GFP and ß-catenin antibodies with DAPI. (*f*) Montage of images taken from supplemental video 2, showing differential interference contrast (DIC) and ß-catenin-mEGFP expression over 6 h. *Closed and open triangles* indicate ß-catenin-mEGFP signals Off and ON afterward, respectively. *Scale bars* 25 μm (*b*, *e*, *f*).

*Wnt downstream mediator*, *ß*-*catenin expressed heterogeneously in ESCs*. Because ß-catenin is an important mediator to activate Wnt target genes in nucleus (Merrill 2012), we then examined the ß-catenin level, prior to SFEBq differentiation, namely ESC state (Fig. 3*a*). Notably, by detecting endogenous ß-catenin protein, we found cells possessing nuclear ß-catenin signals are partial population, indicating ß-catenin level in ESC state may be highly heterogeneous or low (Fig. 3*b* and video 1). Heterogeneous activity of endogenous Wnt signaling in individual ESCs was also seen in previous report showing that TCF-GFP reporter was wide range in expression via FACS analysis (Blauwkamp *et al.*^2012^). Next, to observe ß-catenin expression dynamics in living cells, we established knock-in line with ß-catenin::mEGFP under control of CAG promoter in Rosa26 locus in which exogenous genes stably expressed throughout development (Soriano 1999; Abe *et al.*^2011^) (Fig. 3*c*, *d*; see “Materials and Methods” section). Then, we evaluated the expression and localization of ß-catenin::mEGFP by immunohistochemistry and confirmed ß-catenin::mEGFP signals were comparable to endogenous ß-catenin signals, indicating this knock-in line faithfully represented the ß-catenin signals (Fig. 3*e*; see Fig. 3*b* for comparison). Subsequently, we observed dynamic ß-catenin::mEGFP signals in live condition and each cell in a colony was heterogeneous in expression dynamics of ß-catenin::mEGFP signals (Fig. 3*f* and video 2). This is consistent with previous report, showing ß-catenin fluctuation in ESCs (Marucci *et al.*^2014^).

**Figure 4.**
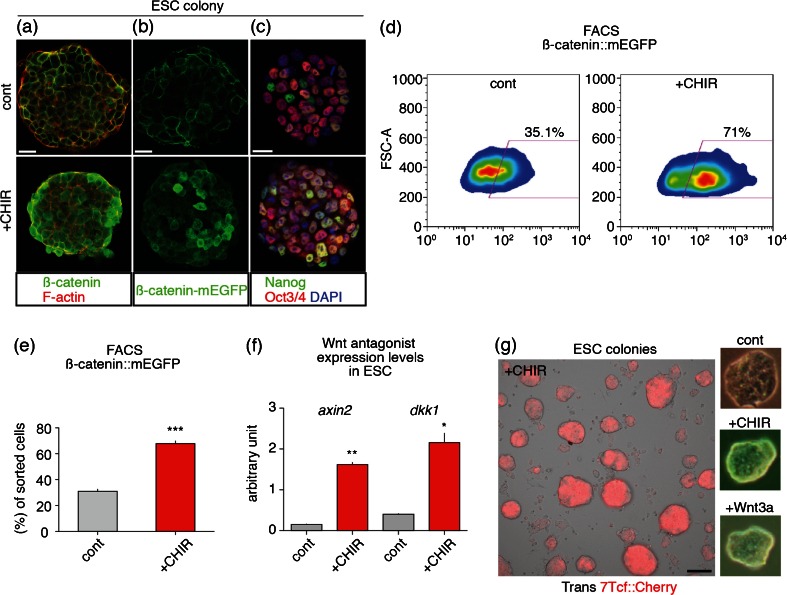
Activation of Wnt signaling increased ß-catenin signals and Wnt target gene expressions. (*a*–*c*) Immunohistochemistry was performed on control and CHIR-treated (for 48 h) ESC colonies using ß-catenin with phalloidin (*a*), GFP (*b*), Nanog and Oct3/4 (*c*) antibodies. *Scale bars* 25 μm. (*d*) FACS analysis of ß-catenin-mEGFP line. (*e*) Quantification of ß-catenin-mEGFP positive cells by FACS. (*f*) Quantification of *axin2* and *dkk1* expression via RT-qPCR, following CHIR treatment for 48 h. *Error bars* indicate standard error of the mean of each experiment. (*g*) Merged images of transillumination (Trans) and 7Tcf::Cherry signals of ESC colonies by addition of 10 μM CHIR for 48 h. *Scale bar* 100 μm. Transillumination images of single ESC colony in control, CHIR- (10 μM) and Wnt3a- (200 ng/ml) treated condition (for 48 h).

*Activation of Wnt signaling in ESCs promoted rostral forebrain differentiation*. To reduce heterogeneous (or low) ß-catenin signals in each cell, we then tested activation of Wnt signaling in ESC state by addition of 10 μM CHIR, which inhibits GSK3ß to upregulate Wnt signaling through inhibition of ß-catenin degradation (Murray *et al.*^2004^; Clevers 2006; Bain *et al.*^2007^). As compared with control condition, CHIR-treated cells highly expressed ß-catenin in nucleus in each cell (Fig. 4*a*, *b*). In addition, CHIR treatment did not substantially change the pluripotent marker (Niwa 2007; Nichols and Smith 2012), Nanog and Oct3/4 expressions (Fig. 4*c*). Notably, we found that strong ß-catenin-expressing population significantly increased in CHIR condition by FACS analysis (Fig. 4*d*, *e*). Next, to confirm the upregulation of Wnt target genes, we performed RT-qPCR analysis and found CHIR treatment strongly increased expression of Wnt antagonist, *axin2*, and *dkk1* as target genes (Jho *et al.*^2002^; Niida *et al.*^2004^) (Fig. 4*f*). We also observed strong expression of 7Tcf::Cherry in ESCs by addition of CHIR (Fig. 4*g*). Furthermore, compact colony morphology was seen in CHIR-treated condition, reminiscent of recombinant Wnt3a-treated condition (Fig. 4*g*).

These results indicate that activation of Wnt signal directly upregulates ß-catenin in nucleus expression and Wnt antagonists, which then have a potential role for inhibiting Wnt signaling (Kawano and Kypta 2003).

**Figure 5.**
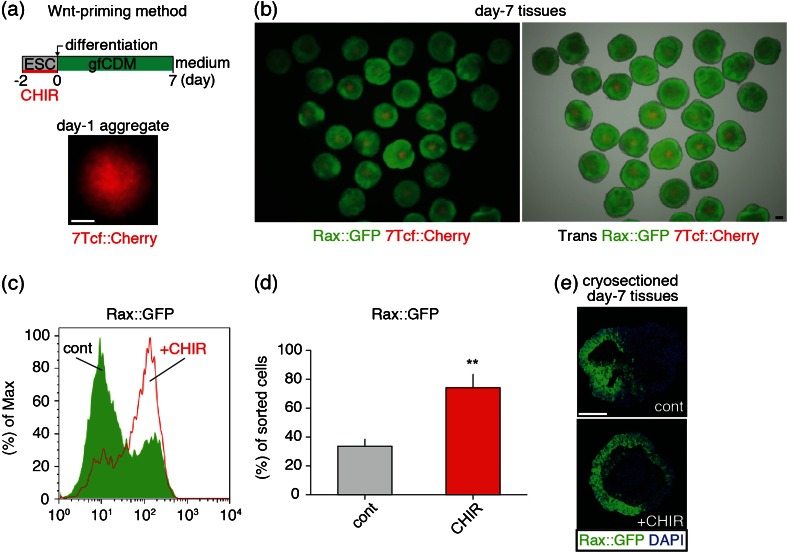
Wnt-priming method promotes rostral forebrain differentiation. (*a*) Diagram of Wnt-priming method. Day-1 aggregate in this method shows 7Tcf::Cherry signals. (*b*) Day-7 tissues showing transillumination (Trans), Rax::GFP and 7Tcf::Cherry signals. (*c*) FACS analysis of CHIR-treated day-7 cells compared with control day-7 cells. Wnt-priming method was not used in control condition. (*d*) Quantification of Rax::GFP positive cells by FACS. (*e*) Cryosectioned day-7 tissues, showing Rax::GFP signals with DAPI for counter staining. *Scale bars* 100 μm (*a*, *b*, *e*).

Finally, we tested the efficacy of rostral forebrain differentiation in CHIR-treated condition (hereafter called, Wnt-priming method) to stabilize ß-catenin and Wnt^+^ populations during ESC maintenance (Fig. 5*a*). Surprisingly, we found SFEBq day-7 tissues has clear and strong induction of Rax::GFP^+^ rostral forebrain differentiation (Fig. 5*b*; see Fig. 1*b* for comparison). In addition, 7Tcf::Cherry signals at day 1 reduced at day 7 (Fig. 5*a*, *b*). FACS analysis showed also CHIR-treated ESCs preferentially differentiated into Rax^+^ cells, compared with control condition (Fig. 5*c*, *d*). By immunostaining following cryosection of tissues, we confirmed that Wnt-priming method promoted Rax::GFP signals of tissue with clear epithelial structure (Fig. 5*e*).

Although, Wnt inhibition in ESC differentiation promote rostral neural fate (Haegele *et al.*^2003^; Nasu *et al.*^2012^), these results raised a possibility that Wnt antagonists in Wnt-priming method minimized Wnt signaling during rostral forebrain tissue development in vitro. However, future work requires how Wnt antagonists act during in vitro rostral forebrain differentiation in this culture system.

## Conclusions

Research of the relationship between Wnt/ß-catenin and rostral forebrain lineage using our results will provide useful insights into mechanisms of Wnt/ß-catenin signaling in ESC culture system for the future improvement of regenerative medicine.

## Electronic supplementary material

Below is the link to the electronic supplementary material.

Video 1 Z-scanning of ß-catenin expression along the apical-basal of ESC colony, related to Fig. 3 *b. Left*, ß-catenin (*green*); *right*, ß-catenin and DAPI (*magenta*) were observed by confocal microscope. The movie was taken at 1 frame per 0.5 μm step and played at 15 fps. (MP4 4442 kb)

Video 2 Dynamics of ß-catenin-mEGFP expression in ESC colony, related to Fig. 3 *f. Left*, ß-catenin-mEGFP; *right*, DIC were observed by time-lapse imaging. The movie was taken at 1 frame per 15 min and played at 15 fps. (MP4 7882 kb)
